# Tröger’s Base Polyimide Membranes with Enhanced Mechanical Robustness for Gas Separation

**DOI:** 10.3390/polym17040524

**Published:** 2025-02-18

**Authors:** Xingfeng Lei, Zixiang Zhang, Yuyang Xiao, Qinyu Yu, Yewei Liu, Xiaohua Ma, Qiuyu Zhang

**Affiliations:** 1 Xi’an Key Laboratory of Functional Organic Porous Materials, School of Chemistry and Chemical Engineering, Northwestern Polytechnical University, Xi’an 710072, China; 2 Key Laboratory of Special Functional and Smart Polymer Materials of Ministry of Industry and Information Technology, School of Chemistry and Chemical Engineering, Northwestern Polytechnical University, Xi’an 710072, China; 3 Key Laboratory of Material Physics and Chemistry Under Extraordinary Conditions of Ministry of Education, School of Chemistry and Chemical Engineering, Northwestern Polytechnical University, Xi’an 710072, China; 4 State Key Laboratory of Separation Membranes and Membrane Processes, National Center for International Joint Research on Membrane Science and Technology, Tiangong University, Tianjin 300387, China

**Keywords:** gas separation, polyimide, Tröger’s base, mechanical robustness, membrane

## Abstract

The rigid V-shaped Tröger’s base (**TB**) unit has been proven efficacious in creating microporosity, making **TB**-based polyimides (PIs) exhibiting significant advantages in simultaneously increasing gas permeability and selectivity for the separation industry. However, **TB**-based PIs commonly display undesired mechanical performance due to the low molecular weight resulting from the evident steric hindrance and low reactivity of **TB**-containing diamines. Herein, a novel diamine-containing bisimide linkage (**BIDA**) has been synthesized and then polymerized with paraformaldehyde via a moderate “**TB** polymerization” strategy to furnish polymers simultaneously, including imide linkages and **TB** units in the polymer main chains, namely, **TB-PI**s. This **TB** polymerization strategy avoids the direct polymerization of dianhydride with low-reactivity **TB** diamine. After incorporating a meta-methyl substituent into **BIDA** diamine, the *m*-**MBIDA** diamine-derived *m*-**MTBPI** ultimately exhibits a high molecular weight, good tensile strength (90.4 MPa) and an outstanding fracture toughness (45.1 MJ/m^3^). And more importantly, the *m*-**MTBPI** membrane displays an evidently enhanced gas separation ability in comparison with **BIDA**-derived **TBPI**, with overall separation properties much closer to the 1991 Robeson upper bound. Moreover, no sign of plasticization appears for the *m*-**MTBPI** membrane when separating a high-pressure CO_2_/CH_4_ mixture (*v*/*v* = 1/1) up to 20 bar, with the CO_2_/CH_4_ mixed-gas separation performance approaching the 2018 upper bound.

## 1. Introduction

Membrane-based gas separation technology is a new type of gas separation method with high separation efficiency, low energy consumption and simple operation. It plays an essential role in many fields of industrial crude material pre-treatment and product post-treatment procedures, such as natural gas purification, hydrogen recovery and the enrichment of olefin and alkyne. For example, membranes with favorable CO_2_/CH_4_ separation selectivity can be applied to the purification of natural gas by removing carbon dioxide before combustion to reduce carbon emissions and increase the heating value [1].

An organic polymeric membrane is widely used in industrial gas separation thanks to its advantages, like excellent plasticity, high selectivity, better formability and processability, lower production cost, low separation energy consumption, etc. [2,3,4,5]. It should be noted that an ideal gas separation membrane needs to combine high selectivity, high permeability and desirable mechanical properties to balance the needs of industrial production [2,6,7]. In recent decades, numerous polymeric membranes have been synthesized and successfully applied in practical production for their superior properties, such as cellulose, polysulfone, polyaryl ether or polyimide [8]. However, for polymeric membranes, there is a “trade-off” relationship between its gas permeability and gas pair selectivity. Any attempt to increase the permeability of the membrane will inevitably reduce the gas pair selectivity, and vice versa. In 1991, Robeson put forward the “Robeson upper bound” between the permeability and selectivity of polymer membranes [9] and then revised and updated it in 2008 [10]. Pinnau et al. renewed the upper bound of permeability and selectivity for the O_2_/N_2_, H_2_/N_2_ and H_2_/CH_4_ gas pairs in 2015 [11]. These experiential upper bounds are widely recognized and become a criterion for evaluating the gas separation performance of membranes.

Polyimide (PI) is a type of polymer that contains an imide ring in the polymer main chain, which is well known for its exceptional thermal stability, excellent mechanical properties, great solvent resistance and chemical stability. PIs are often used as gas separation membrane materials not only because they can work under harsh conditions but also exhibit prominent ability to screen and separate different gas molecules like CO_2_/CH_4_ and CO_2_/N_2_ at a high separation coefficient [12,13,14]. Matrimid 5218 is one of the most widely used commercial polyimides. Its twisted molecular structure increases its backbone rigidity and reduces the stacking efficiency of polymer chains, making Matrimid 5218 exhibit a more attractive gas separation performance compared to regular polymers like cellulose, but its performance is still far from satisfactory. So, it is practical to introduce shape-persistent units into the polymer backbone to enhance polymer rigidity for selectivity and increase the fractional free volume (*FFV*) for permeability at the same time.

Like the “trade-off” effect between gas permeability and selectivity, it is difficult to reconcile membrane’s high gas separation ability with good mechanical strength [15,16,17,18,19,20]. Loosely packed and highly twisted polymer chains often result in weak intermolecular interactions, and such highly permeable membranes tend to have a rich micropore structure, making it even more difficult to exhibit good mechanical properties [21,22,23,24,25]. But it is undeniable that the gas separation industry always favors a stable and reliable product. For example, the industrial separation membrane Matrimid 5218 has a tensile strength of 72.3 MPa and an elongation at break of 19.4% [26], while the highly permeable **PIM-1** only exhibits a tensile strength below 50 MPa and an elongation at break of 10.2–15.8% [25], which is unsatisfactory for a long-term high-pressure separation process. So improving the mechanical properties of highly permeable separation membranes is necessary and essential for industrial gas separation membranes and their modules [27].

Among a few twisted molecular elements reported thus far, Tröger’s base (**TB**), a non-co-planar and quite rigid V-shape bridged bicyclic linking group containing two tertiary amine units, has been proven efficacious in creating microporosity [21,23,28]. Inspired by the effectiveness of **TB** moiety for constructing PIMs, in the past few decades, **TB** unit has been integrated into the polyimide backbone to produce highly permeable PIs with intrinsic microporosity, that is, **PIM-TB-PI**s. Guiver [27], Lee [29], McKeown [21] and other researchers [5,30,31] reported the fabrication and gas transport properties of **PIM-TB-PI**s. Yan et al. [32] reported an intrinsically microporous polyimide by polymerizing 3,3′-di-tert-butyl-2,2′-dimethoxy-[1,1′-binaphthalene]-6,6′,7,7′-tetracarboxylic dianhydride (**TNTDA**) with **TBDA1** diamine containing the **TB** unit, offering a **PIM-TB-PI** membrane showing enhanced *FFV* (0.181), a high BET surface area (379 m^2^ g^−1^) and favorable O_2_/N_2_ separation performance. However, the loosened chain packing weakens the chain–chain interaction, resulting in the tensile strength being only 70 MPa and the elongation at break merely 5.3% [32]. Lee et al. [29] synthesized two **TB**-containing PIs (**Bio-TBPI-1** and **Bio-TBPI-2**) by reacting two rigid bio-based anhydrides (**MMDA** and **FDDA**) with one **TB** diamine, further improving the BET surface area and *FFV* of **TB**-containing PIs to ~500 m^2^ g^−1^ and ~0.2. Both **Bio-TBPI-1** and **Bio-TBPI-2** display excellent gas separation performance and exceed the 1991 Robeson upper bound. However, the tensile strength of the two membranes falls into the range of 76.3–82.6 MPa with the elongation at break of 7.1–8.7% [29].

**Scheme 1:**
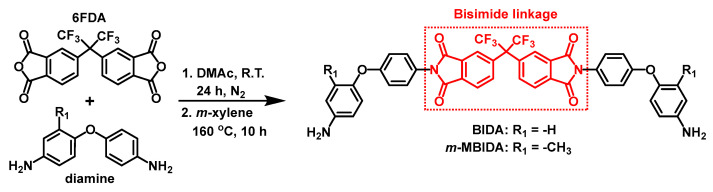
Reaction route for the synthesis of diamine monomers containing bisimide linkage.

After summarizing the above investigations, we can conclude that, for the preparation of **TB**-based **PIM-PI**s, usually a judiciously designed diamine monomer containing **TB** units is synthesized and then polymerized with a twisted dianhydride, such as 4,4′-(hexafluoroisopropylidene)-diphthalic anhydride (**6FDA**) and **TNTDA** [29,33,34]. However, the considerable rigidity and steric hindrance of the **TB** unit impede the nucleophilic attack of the amino group on the anhydride group. Thus, **TB**-based diamine usually exhibits undesired polymerization reactivity when directly polymerizing **TB**-based diamine with dianhydride, eventually leading to the low molecular weight of polymers and hence the poor mechanical strength along with the compromised toughness (robustness) of the polymer membranes. To increase the molecular weight of **TB**-containing polyimides, the polymerization is usually accomplished in *m*-cresol via a typical “one-pot” method at a fairly high temperature (e.g., 180–220 °C), and sometimes, a co-diamine monomer with higher reactivity has to be introduced to increase the molecular weight [35]. To avoid the low reactivity of **TB**-based diamine, herein, a novel diamine-containing bisimide linkage (**BIDA**) has been synthesized (Scheme 1) and then the **BIDA** is allowed to react with paraformaldehyde in the solvent of trifluoroacetic acid (TFA) to simultaneously introduce the imide linkages and **TB** units in the polymer main chains. That is, we make use of the “**TB** polymerization” strategy, which was first reported by the McKeown group in 2013 [21], to construct **PIM-TB-PI**s while avoiding direct polymerization of dianhydride with low-reactivity **TB** diamine. To investigate the effect of the substituent on the molecular weight and final separation performance of the resulting PIs, a meta-methyl substituent to the amino group is introduced to provide *m*-**MBIDA**. The resulting two polyimides exhibit desirable molecular weight, high thermal stability and, especially for *m*-**MBIDA**-derived *m*-**MTBPI**, good tensile strength (90.4 MPa) and outstanding fracture toughness (45.1 MJ/m^3^). *m*-**MTBPI** displays a more desirable gas separation capability in comparison with **BIDA**-derived **TBPI**, whose overall separation properties are much closer to the 1991 Robeson upper bound for the CO_2_/CH_4_, CO_2_/N_2_, H_2_/CH_4_ and O_2_/N_2_ gas pairs upon single-gas tests. Moreover, *m*-**MTBPI** exhibits good plasticization resistance when separating the high-pressure CO_2_/CH_4_ mixture (*v*/*v* = 1/1) up to 20 bar, with the CO_2_/CH_4_ mixed-gas separation performance approaching the 2018 upper bound.

## 2. Materials and Methods

The Materials and Measurement details have been provided in the Supplementary Materials.

### 2.1. Synthesis of Bisimide Diamine Monomers

*Synthesis of BIDA.* A total of 7.509 g of 4,4′-diaminodiphenylether (ODA) and 5.553 g of 4,4′-(hexafluoroisopropylidene)diphthalic anhydride (6FDA) were weighed in a three-necked round-bottom flask. Then, 50 g of anhydrous DMAc was added under a nitrogen atmosphere and the reaction was stirred at room temperature for 24 h. Thereafter, 16 mL of *m*-xylene was added to the mixture and the reaction system was heated to reflux, during which the water evolved was removed by refluxing the azeotrope in a Dean–Stark trap. After refluxing for 10 h, most of the *m*-xylene was removed and the reaction system was allowed to cool to room temperature. The reaction solution was poured into distilled water and the solids were precipitated out. The crude products were collected by vacuum filtration and thoroughly washed with deionized water. After recrystallization 2–3 times in a mixture of ethanol/water (*v*/*v* = 3:1), the BIDA was obtained as a bright-yellow powder in ~65% yield (6.582 g). FT-IR (KBr, 25 °C): 3464–3364 cm^−1^ (N-H stretching), 3044–2881 cm^−1^ (aromatic and aliphatic C-H stretching), 1784 cm^−1^ (imide C=O asymmetric stretching), 1722 cm^−1^ (imide C=O symmetric stretching), 1381 cm^−1^ (imide C-N stretching), 1240 cm^−1^ (C-O asymmetric stretching) and 721 cm^−1^ (imide C=O bending).

*Synthesis of m-MBIDA. m*-MBIDA was synthesized using the same procedure as that for BIDA, except that *m*-MODA was used instead of ODA. Yield: ~56%. FT-IR (KBr, 25 °C): 3470–3376 cm^−1^ (N-H stretching), 1774 cm^−1^ (imide asymmetric C=O stretching), 1724 cm^−1^ (imide C=O symmetric stretching), 1381 cm^−1^ (imide C-N stretching), 1234 cm^−1^ (C-O asymmetric stretching) and 723 cm^−1^ (imide C=O bending).

### 2.2. Preparation of Tröger’s Base-Based Polyimides (TBPIs)

**Scheme 2:**
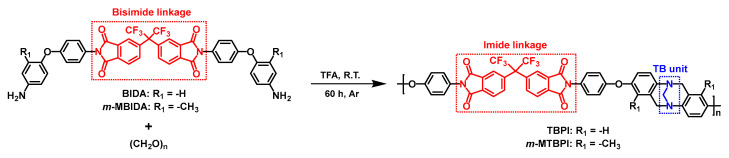
Reaction route for the preparation of TB-based polyimides.

*Preparation of TBPI.* A 100 mL Schlenk flask containing BIDA and paraformaldehyde in a mole ratio of 1:4.5 was vacuumized and backfilled with argon three times to create an anhydrous and oxygen-free environment. Afterwards, trifluoroacetic acid (TFA) was slowly injected into the flask at 0 °C under an argon flow to adjust the solid content to 10–15 w.t.%. After stirring at room temperature for 60 h in an argon atmosphere, a viscous polymer solution was obtained (Scheme 2). The reaction solution was then slowly poured into deionized water for precipitation and then ammonium hydroxide was slowly added to adjust the pH to 7–8. After filtration and thorough washing with deionized water, the polymer precipitates were dissolved in chloroform and then subject to vacuum filtration. The polymer solution was then precipitated in methanol and this dissolution–precipitation process was repeated three times to completely remove the solvent residuals and any insoluble impurities. Finally, fibrous polyimides were obtained in ca. 95% yield after vacuum drying at 100 °C for 24 h. FT-IR (KBr, 25 °C): 3070–2848 cm^−1^ (aromatic and aliphatic C-H stretching), 1784 cm^−1^ (imide C=O asymmetric stretching), 1726 cm^−1^ (imide C=O symmetric stretching), 1249 cm^−1^ (Tröger’s base C-N stretching), 1099 cm^−1^ (C-F stretching) and 723 cm^−1^ (imide C=O bending). GPC results (THF, 0.5 mL min^−1^, 25 °C): *M*_n_ = 12,658 g·mol^−1^, *M*_w_ = 35,703 g·mol^−1^ and *M*_w_/*M*_n_ = 2.82.

*Preparation of m-MTBPI. m*-MTBPI was prepared using the same procedure as that for TBPI, except that *m*-MBIDA was used instead of BIDA. Yield: ~96%. FT-IR (KBr, 25 °C): 2950–2850 cm^−1^ (aromatic and aliphatic C-H stretching), 1786 cm^−1^ (imide C=O asymmetric stretching), 1728 cm^−1^ (imide C=O symmetric stretching), 1255 cm^−1^ (Tröger’s base C-N stretching), 1084 cm^−1^ (C-F stretching) and 723 cm^−1^ (imide C=O bending). GPC results (THF, 0.5 mL min^−1^, 25 °C): *M*_n_ = 29,320 g·mol^−1^, *M*_w_ = 65,503 g·mol^−1^ and *M*_w_/*M*_n_ = 2.32.

### 2.3. Preparation of Polyimide Membranes

A total of 0.5 g of polymer was well dissolved in 10 mL of DMF (solid content: ~5 w.t.%) in a vial at room temperature. The solution was then filtered by using a nylon filter (pore size 0.22 μm) and cast onto a flat, dust-free Petri dish (diameter of 9 cm) to slowly evaporate the solvent at 60 °C for 48 h. It was further heated at 160 °C under high vacuum for 12 h to yield self-standing membranes with a thickness of ~45 μm as displayed in Figure S1 (Supplementary Materials). Before the gas permeation test, the two membranes were Soxhlet-extracted by methanol for 24 h to eliminate physical aging for glassy polymers and completely remove the solvent residuals. Afterwards, the membranes were directly subject to ambient air drying for 24 h followed by vacuum degassing at 35 °C for 24 h.

## 3. Results and Discussion

### 3.1. Structural Characterization

**Figure 1:**
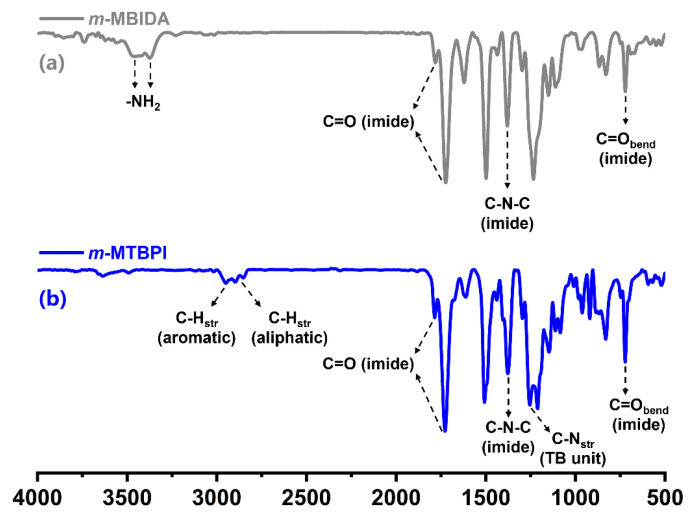
FT-IR spectra of (**a**) *m*-**MBIDA** diamine and (**b**) *m*-**MTBPI** polymer.

**Figure 2:**
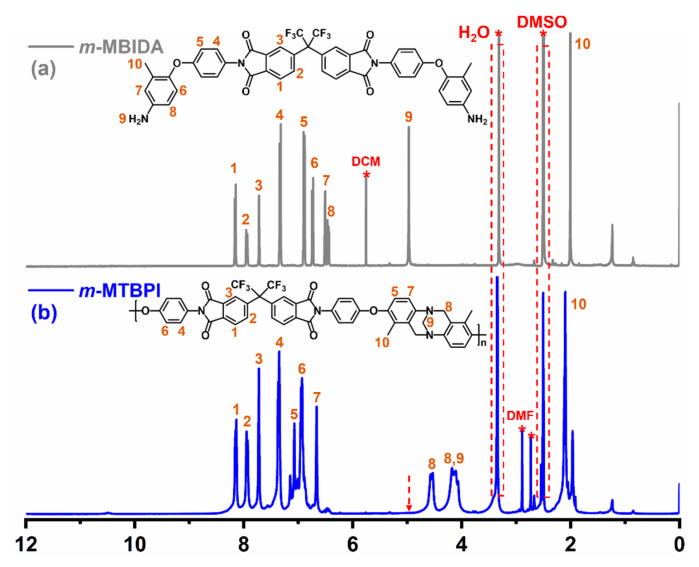
^1^H-NMR spectra of (**a**) *m*-**MBIDA** diamine and (**b**) *m*-**MTBPI** polymer in DMSO-*d*_6_. The asterisk indicates solvent and moisture residuals or the *H*-grease signal.

The molecular structures of *m*-**MBIDA** diamine and *m*-**MTBPI** polymer were characterized by FT-IR and nuclear magnetic resonance (NMR) spectroscopy techniques. As shown in Figure 1a, the characteristic absorption bands for the primary amine of *m*-**MBIDA** appear at 3470–3376 cm^−1^. After **TB** polymerization, the signal for N-H stretching almost vanishes, while the C-N stretching located at 1255 cm^−1^ and attributed to the **TB** unit appears (Figure 1b), suggestive of a high monomer conversion and successful **TB** polymerization. The typical absorption bands for the five-membered imide ring, respectively, at ca. 1375 cm^−1^ assigned to imide C-N stretching, ca. 1780 cm^−1^ and 1725 cm^−1^ attributed to imide C=O asymmetric and symmetric stretching and ca. 720 cm^−1^ due to imide C=O bending, can be found in both *m*-**MBIDA** and *m*-**MTBPI** polymer, demonstrating that the imide ring is intact during **TB** polymerization and the *m*-**MTBPI** polymer simultaneously contains the imide structure and **TB** unit. Additionally, the FT-IR spectra also contain the information for C-F stretching at ca. 1099 cm^−1^, C-O-C stretching at 1235 cm^−1^ and C-H stretching (aromatic and aliphatic) at 3020–2850 cm^−1^. Figure 2 gives the ^1^H-NMR spectra of *m*-**MBIDA** and *m*-**MTBPI** polymer. The hydrogen of the meta-methyl substituent for *m*-**MBIDA** is located at 2.01 ppm, while that for *m*-**MTBPI** polymer resonates at 1.96–2.09 ppm. The proton that appears at 4.97 ppm is assigned to the amino group, while it completely vanishes after polymerization as indicated in Figure 2b. In addition, the aliphatic hydrogens (-CH_2_-) within the V-shaped **TB** unit are split into multiple peaks and can be found at 4.06–4.17 ppm and 4.52–4.56 ppm. The other protons can also be fully assigned, and the hydrogen concentrations (integral area) are consistent with the theoretical composition. Similar results can also be identified in the FT-IR and NMR spectra for **BIDA** and **TBPI** polymer shown in Figures S2 and S3 (Supplementary Materials). Notably, the chemical shift of the amino group for **BIDA** is 5.05 ppm, slightly larger than that of *m*-**MBIDA** (4.97 ppm), implying a decreased amino reactivity in the order of **BIDA** < *m*-**MBIDA**. All these results confirm the well-defined molecular structures of bisimide diamines and the successful preparation of **TB**-based polyimides.

### 3.2. Thermal Properties of the Two Membranes

**Figure 3:**
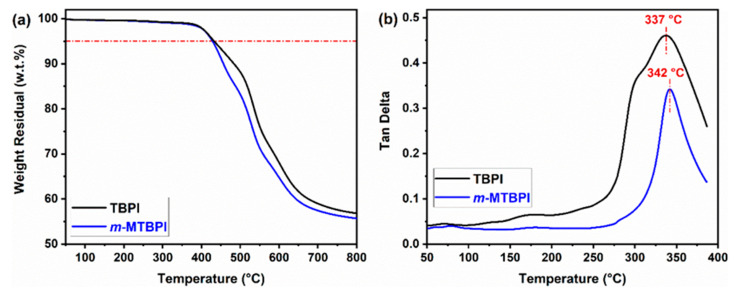
(**a**) TGA curves and (**b**) DMA curves of **TBPI** and *m*-**MTBPI** membranes. *Note:* the red dotted line in (**a**) indicates 95 w.t.% weight residuals.

The thermal properties of the two membranes were evaluated by DMA and TGA. The results are shown in Table 1 and Figure 3. Both **TBPI** and *m*-**MTBPI** have desirable thermal stability. The 5% and 10% weight loss temperatures (*T*_d5_ and *T*_d10_) of **TBPI** are, respectively, 432 °C and 483 °C, while those for *m*-**MTBPI** are a little inferior, respectively, 428 °C and 456 °C, most likely due to the introduction of the labile methyl group. The methyl substituents may pyrolyze into benzyl radicals upon heating and hence cause defects to the polymer backbone [36], which probably accelerates thermal decomposition and leads to a lower char yield (55.7 w.t.%) in comparison with that of **TBPI** (56.8 w.t.%). However, the meta-methyl substituent may have a role in restricting the rotation of the diphenyl ether linkage (C-O-C) and thus increases the chain rigidity, ultimately giving rise to a little higher *T*_g_ (342 °C), as shown in Figure 3b and Table 1. In addition, the higher molecular weight of *m*-**MTBPI** would strengthen the cohesive force of the polymer network and this may inhibit the segmental motions and increase the *T*_g_.

### 3.3. Mechanical Properties of the Two Membranes

**Figure 4:**
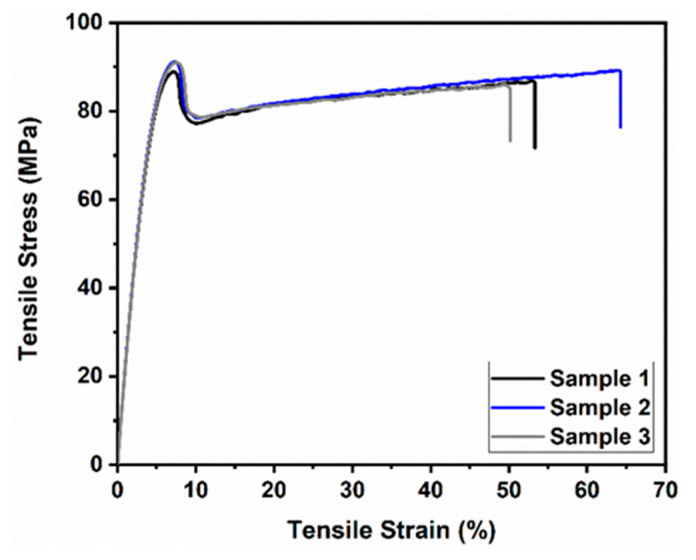
Tensile stress–strain curves of *m*-**MTBPI** membranes.

Since the **TBPI** membrane was brittle and could not survive the tensile test, only the *m*-**MTBPI** membrane was measured for tensile properties. From Table 2, the tensile strength of the *m*-**MTBPI** membrane is as high as 90.4 MPa, in combination with a tensile modulus up to 2.27 GPa, which is indicative of good mechanical robustness. Notably, the elongation at break of the *m*-**MTBPI** membrane is surprisingly up to 55.9%, with the static toughness as high as 45.1 MJ/m^3^, and its tensile stress–strain curves (Figure 4) display an evident ductile fracture manner, which is quite rare for gas-permeable membranes. Additionally, the flexibility outperforms many of the **PIM-PI**s and a number of the peer competitors as well as a few conventional commercial polymers, whose elongation at break is typically 4–8% coupled with a tensile strength commonly in the range of 50–70 MPa [32,35,37,38]. The desirable mechanical strength and outstanding fracture toughness make *m*-**MTBPI** highly resistant to stress fatigue and promising for gas separation applications.

### 3.4. Microporosity of the Two Membranes

**Figure 5:**
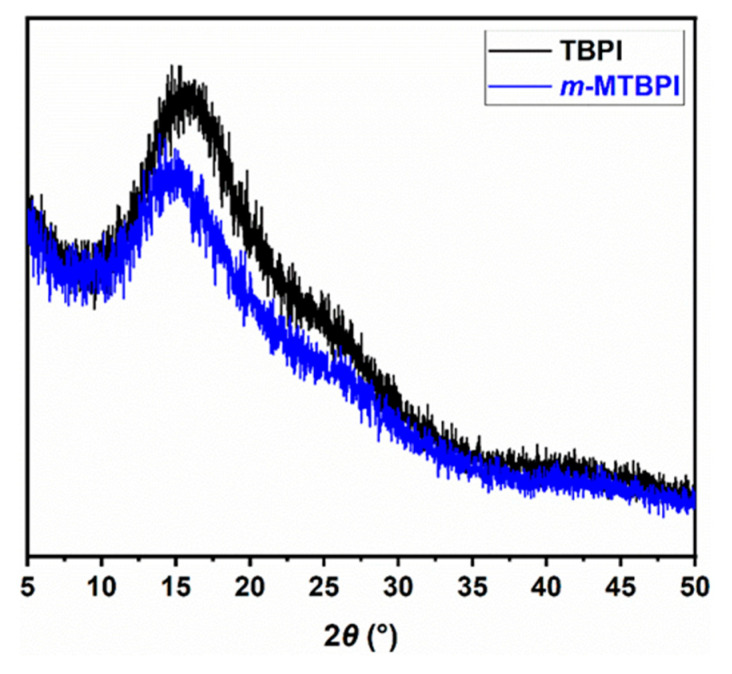
WAXD patterns of **TBPI** and *m*-**MTBPI** membranes.

The aggregation state of the two membranes was investigated by wide-angle X-ray diffraction (WAXD) and the *d*-spacing was calculated according to Bragg’s law. As is indicated by the WAXD diffraction patterns shown in Figure 5, it can be concluded that the two membranes are amorphous in nature. In comparison with **TBPI**, *m*-**MTBPI** exhibits larger *d*-spacing (6.08 Å vs. 5.66 Å), implying an enlarged average segmental distance and looser interchain packing within the *m*-**MTBPI** membrane, which is also consistent with its lower density listed in Table 3. The above results prove that the introduction of the meta-methyl substituent loosens the interchain packing and enhances the free volume fraction of the *m*-**MTBPI** membrane, which contributes to gas permeation.

**Figure 6:**
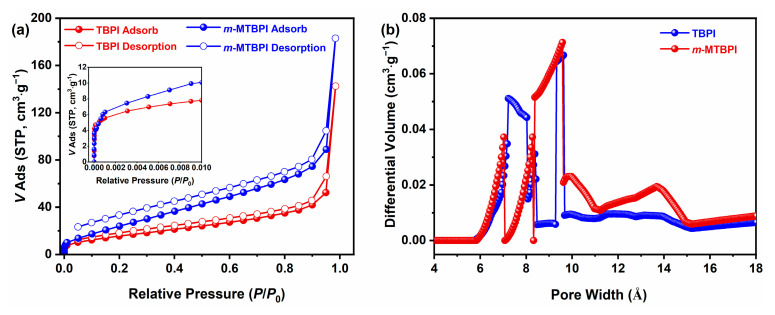
(**a**) N_2_ adsorption and desorption isotherms of the resulting polyimides tested at 77 K; (**b**) pore-size distribution of the resulting polyimides calculated by the H-K method according to N_2_ sorption isotherms.

The microporosity of the two polyimides were analyzed by N_2_ physisorption at 77 K and the results are provided in Figure 6 and Table 4. From the physisorption isotherms (Figure 6a), we can see that, in contrast to **TBPI**, *m*-**MTBPI** not only shows more rapid N_2_ adsorption at a very low relative pressure (*P*/*P*_0_ < 0.01) but also exhibits more pronounced N_2_ uptake all through the testing pressure range. This suggests that the pores within *m*-**MTBPI** are more abundant than those within **TBPI**. The pore-size distribution derived from the H-K method is depicted in Figure 6b. It is clearly indicated that *m*-**MTBPI** possesses a certain amount of “larger” micropores (1.0–1.5 nm), and the pores in the range of 0.8–1.0 nm are more remarkable than **TBPI**. The more abundant “larger” micropores in *m*-**MTBPI** would contribute to the fast diffusion of gas molecules, while **TBPI** has more micropores in the range of 0.7–0.8 nm, which almost lie in the ultra-microporosity range (<7 Å) and are essential for increasing the permselectivity. The BET surface areas of **TBPI** and *m*-**MTBPI** are, respectively, 59.8 m^2^·g^−1^ and 104.5 m^2^·g^−1^, once again proving that the introduction of the meta-methyl substituent inhibits single-bond rotation and enhances the rigidity of the polymer chains, offering more interchain free volume and more micropores.

### 3.5. Gas Separation Properties

After the micropore structure was identified, we next explored the gas separation performance of the two PI membranes. Before the test, both **TB**-based **PI** membranes were treated with the Soxhlet extraction protocol to ensure the complete removal of trace amounts of the solvent residue and low-molecular-weight oligomers. The overall gas separation performance of the two **TB**-based **PI**s and its unpretreated control group are compared in Figure S5 (Supplementary Information). It is clearly indicated that the gas permeability of the two **TB**-based **PI** membranes after Soxhlet extraction is evidently improved compared with the unpretreated control samples. And the permselectivity for *m*-**MTBPI** is also increased for all the tested gas pairs after Soxhlet extraction, reflecting that the solvent residual absorbed in the polymer network indeed exerts remarkable negative effects on the overall gas separation properties. The gas permeability *P* (Barrer) and the ideal permselectivity (*α*) for the Soxhlet-extracted membranes are listed in Table 4. **TBPI** shows moderate gas permeability and gas pair selectivity. After the meta-methyl substituent is introduced, the gas permeability of *m*-**MTBPI** is evidently improved. More specifically, the gas permeability of He, H_2_ and CO_2_, respectively, is 50.4%, 84.1% and 60.6% higher than that of **TBPI**. The gas permeability *P* is calculated according to *P* = *D*·*S* and hence is affected by both the solubility coefficient (*S*) and diffusion coefficient (*D*). As is clearly indicated in Table 5, both the *S* and *D* of *m*-**MTBPI** are obviously higher than those of **TBPI**. Owing to the introduction of the meta-methyl group, the loosely packed molecular chains enlarge the interchain space and create more free volume to allow the gas molecules to go through, hence increasing the *D* for all gas species. Meanwhile, the BET surface area of *m*-**MTBPI** is 74.7% higher that of **TBPI** (104.5 m^2^·g^−1^ vs. 59.8 m^2^·g^−1^), which also has a significant role in increasing the gas permeability. The increased BET surface area can provide more binding sites between the polymer network and gas molecules and favors gas molecule adsorption and solution, ultimately leading to the increase in *S*. It is noteworthy that, as in Table 4, the ideal selectivity (*α*) for the H_2_/N_2_ and CO_2_/CH_4_ gas pairs remains almost unchanged, while it displays 17.4% and 31.0% increases, respectively, for the O_2_/N_2_ and CO_2_/N_2_ gas pairs when comparing *m*-**MTBPI** with **TBPI**. The ideal selectivity (*α*) is defined as *α*_A/B_ = *P*_A_/*P*_B_ and *P* is calculated by *P* = *D*·*S*. Hence, *α*_A/B_ can be expressed as *α*_A/B_ = (*D*_A_/*D*_B_)·(*S*_A_*/S*_B_) = *α*_D_·*α*_S_. This is to say that the gas pair selectivity is determined by both diffusion selectivity (*α*_D_) and solubility selectivity (*α*_S_). As shown in Table 6, for the O_2_/N_2_ and CO_2_/N_2_ gas pairs, the *α*_D_ of *m*-**MTBPI** is larger than that of **TBPI**, while the *α*_S_ reduces when comparing *m*-**MTBPI** with **TBPI**. Therefore, the increase in O_2_/N_2_ and CO_2_/N_2_ selectivity is absolutely owing to the enhanced *α*_D_, which is resulting from the loosened interchain packing with the inclusion of the methyl substituent. For the CO_2_/CH_4_ selectivity of *m*-**MTBPI**, the enhancement of *α*_D_ is exactly offset by the decrement of *α*_S_, ultimately leading to an almost unchanged CO_2_/CH_4_ selectivity. For the small gas molecules (H_2_ and He), the *D* and *S* for both membranes are not provided because the permeation time across the membrane (time lag) is too short. Notably, as is compared in Table 4, for the H_2_/CH_4_ and He/CH_4_ gas pairs, when the methyl substituent is incorporated into the **TBPI** polymer network, the ideal selectivity obviously declines. This is because the increase in CH_4_ permeability is much larger than that in He and H_2_, demonstrating that the permeation of a larger gas molecule is more easily accelerated by the increase in free volume. By comparing the results listed in Table 6, we can draw a conclusion that, in comparison with the larger gas species (i.e., CH_4_ and N_2_), the diffusion coefficient of smaller gas molecules (i.e., CO_2_ and O_2_) is more easily enhanced due to the enlarged free volume, while the solubility (affinity) of the less polar CH_4_ and N_2_ within the polymer network is more easily strengthened than polarizable CO_2_ and O_2_ due to the incorporation of the nonpolar methyl substituent, since the methyl substituent introduction can simultaneously improve the fractional free volume and lower the polarity of the *m*-**MTBPI** network.

**Figure 7:**
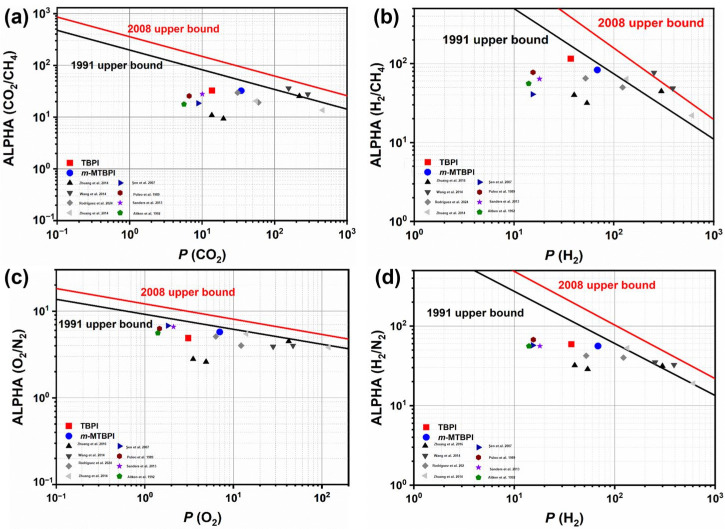
Robeson plots relevant to the resulting polyimide membranes for the (**a**) CO_2_/CH_4_ gas pair, (**b**) H_2_/CH_4_ gas pair, (**c**) O_2_/N_2_ gas pair and (**d**) H_2_/N_2_ gas pair. The data points from the commercial polycarbonate, cellulose acetate, Matrimid 5218, polysulfones and several reported **TB-PI**s are shown for comparison. *Note*: The black line and red line, respectively, indicate the 1991 and 2008 Robeson upper bound.

The overall gas separation performance of the two membranes was evaluated relative to the 1991 and 2008 Robeson upper bound, which is plotted in Figure 7. Comparing with **TBPI**, *m*-**MTBPI** exhibits a more desired gas separation property for the CO_2_/CH_4_, H_2_/CH_4_, O_2_/N_2_ and H_2_/N_2_ gas pairs, and its overall gas separation performance is much closer to the 1991 Robeson upper bound [9]. Additionally, in comparison with several commercially available gas separation membranes (i.e., Matrimid 5218, Polycarbonate, etc.) and a few literature-reported **TB-PI**s (i.e., **PI-TB-4**, **PIM-TB-PI-1**, etc.), *m*-**MTBPI** still displays higher gas permeability as well as comparable or much higher gas pair selectivity (Table 4). For instance, the CO_2_ and H_2_ permeability of *m*-**MTBPI** is ~5.2 and ~4.4 times that of the commercial cellulose acetate, and the O_2_ permeability of *m*-**MTBPI** is ~2.3- and ~2.9-fold higher than commercial Matrimid 5218 and polycarbonate materials. Meanwhile, the ideal permselectivity for the CO_2_/CH_4_ and H_2_/CH_4_ gas pairs is, respectively, 1.28 and 1.07 times that of cellulose acetate and 2.21 times for the O_2_/N_2_ gas pair by comparing *m*-**MTBPI** with **PI-TB-5**. All the above results make *m*-**MTBPI** a promising candidate for natural gas upgrade, hydrogen recovery and oxygen enrichment.

**Figure 8:**
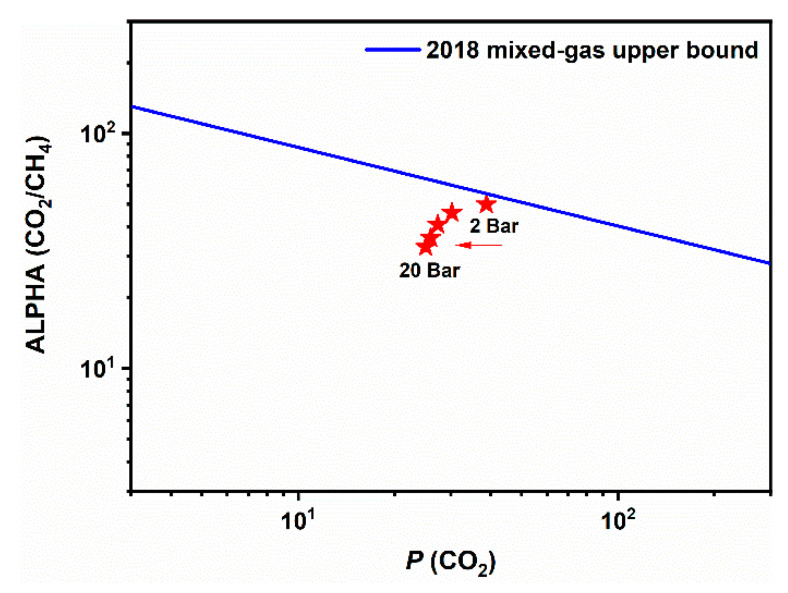
Comprehensive gas separation performance of the *m*-**MTBPI** membrane for the CO_2_/CH_4_ mixed gas (50:50) relative to the 2018 upper bound. The results were obtained in the pressure range of 2–20 bar at 35 °C.

To evaluate the gas separation ability under the simulated industrial conditions, we also tested the mixed-gas (CO_2_/CH_4_ mixture in 50/50 partial pressure) separation property of the *m*-**MTBPI** membrane at 35 °C. The results are shown in Figure 8 and Table 7. It is indicated that the *m*-**MTBPI** membrane exhibits both higher CO_2_ permeability and CO_2_/CH_4_ selectivity at 2 bar than that of the single-gas measurement, suggesting a competitive sorption mechanism in which CO_2_ preferentially adsorbs onto the micropore wall, thereby impeding CH_4_ permeation [43,44]. With the increase in the testing pressure, the CO_2_ permeability along with the CO_2_/CH_4_ selectivity monotonically decreases, and the comprehensive mixed-gas separation performance gradually drifts away from the 2018 CO_2_/CH_4_ mixed-gas upper bound.

**Figure 9:**
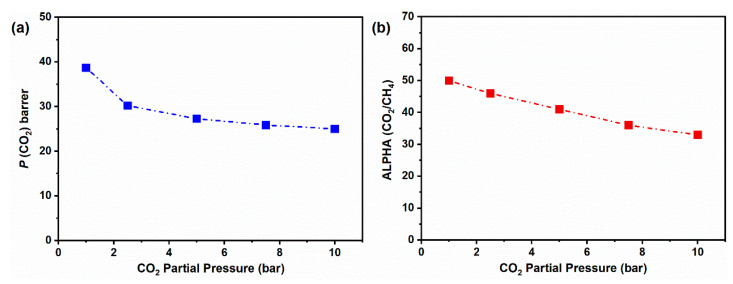
CO_2_/CH_4_ mixed-gas separation performance plotted with increasing upstream pressure for *m*-**MTBPI** membrane: (**a**) CO_2_ permeability and (**b**) CO_2_/CH_4_ gas pair selectivity.

For a couple of the gas separation membranes working under high feed pressure, the polymer network will swell due to the massive dissolution and absorption of condensable gas molecules, for example, CO_2_. This swelling will lead to the expansion of the interchain distance and the increase in fractional free volume, thereby increasing the gas permeability while deteriorating the size-sieving ability and hence reducing the gas pair selectivity [44]. This phenomenon is usually regarded as “plasticization” and a sharp rise in permeability is usually considered as the sign of plasticization of a gas separation membrane, and the very pressure at which the permeability of the membrane starts to rise is called the plasticization pressure [45,46,47]. Therefore, it is crucial to develop membrane materials that can resist plasticization and maintain gas selectivity at high feed pressure [48,49,50]. As shown in Table 7 and Figure 9, when increasing the feed pressure up to 20 bar (CO_2_ partial pressure reaching 10 bar), both the CO_2_ permeability and CO_2_/CH_4_ selectivity of the *m*-**MTBPI** membrane shows a monotonically decreasing trend, and no sign of increase in gas permeability could be observed over the whole pressure range (2–20 bar), which proves that the *m*-**MTBPI** membrane has good plasticization resistance. More importantly, when the feed pressure reaches 20 bar and a CO_2_ partial pressure of 10 bar, the permeability of the *m*-**MTBPI** membrane remains at 25 Barrer along with the selectivity retaining a value of 33, which is still an acceptable result, and thus, the *m*-**MTBPI** membrane can be potentially used for natural gas upgrade.

## 4. Conclusions

In this study, two **TB**-containing polyimides were synthesized through the mild **TB** polymerization strategy by reacting two bisimide-containing diamines with paraformaldehyde. It is indicated that the inclusion of the meta-methyl substituent could not only improve the reactivity of the amino group and chain rigidity but also significantly loosen the interchain distance and increase the microporosity. Hence, *m*-**MTBPI** exhibits a higher molecular weight and a much more abundant microporosity, which make *m*-**MTBPI** exhibit a satisfactory tensile strength (90.4 MPa) coupled with outstanding static toughness (45.1 MJ/m^3^) and desirable thermal properties, including a 5% decomposition temperature of 428 °C and a glass transition temperature of 342 °C. More importantly, *m*-**MTBPI** possesses much better gas separation performance in comparison with **TBPI** and displays good plasticization resistance upon high-pressure separation. Our design concept has put forward a feasible pathway for constructing **TB**-containing PIs for the gas separation industry.

## Figures and Tables

**Table 1:** Thermal properties of **TBPI** and *m*-**MTBPI** membranes.

Samples	*T*_d5_ (°C)	*T*_d10_ (°C)	Char Yield (w.t.%)	*T*_g_ (°C)
**TBPI**	432	483	56.8	337
*m*-**MTBPI**	428	456	55.7	342

**Table 2:** Mechanical properties of *m*-**MTBPI** membrane.

Sample	Tensile Modulus ^a^ (GPa)	Tensile Strength (MPa)	Elongation at Break (%)	Static Toughness ^b^ (MJ/m^3^)
*m*-**MTBPI**	2.27 ± 0.02	90.4 ± 1.4	55.9 ± 8.3	45.1 ± 6.6

^a^ Tensile modulus, regarded as the initial slope for the tensile stress–strain curves. ^b^ Static toughness, calculated by integrating the area under the tensile stress–strain curves.

**Table 3:** The *d*-spacing and density of the **TBPI** and *m*-**MTBPI** membranes.

Samples	*d*-Spacing (Å)	Density (g/cm^3^)
**TBPI**	5.66	1.3851
*m*-**MTBPI**	6.08	1.3347

**Table 4:** Pure gas permeability and ideal permselectivity of **TBPI** and *m*-**MTBPI** membranes as well as a few literature-reported and commercially available separation membranes tested at 35 °C and 2 bar.

Samples	Permeability (Barrer)						Permselectivity (*α*_x/y_)					
	He	H_2_	N_2_	O_2_	CH_4_	CO_2_	H_2_/N_2_	O_2_/N_2_	CO_2_/CH_4_	H_2_/CH_4_	He/CH_4_	CO_2_/N_2_
**TBPI**	39.5	37.1	0.63	3.08	0.32	13.6	58.9	4.89	42.6	115.9	123.4	21.6
*m*-**MTBPI**	59.4	68.3	1.22	7.00	0.82	34.5	56.0	5.74	42.1	83.3	72.4	28.3
**PI-TB-3** [27]	221	299	9.5	42	6.7	218	31.4	4.5	32.7	44.8	33.0	22.9
**PI-TB-4** [27]	37.3	40	1.3	3.5	1.0	13.5	32.1	2.8	13.5	40.0	37.3	10.8
**PI-TB-5** [27]	43.7	53.8	1.9	4.9	1.7	19.6	28.7	2.6	11.5	31.6	25.7	10.5
**TBDA1-6FDA-PI** [23]	199	253	6.5	28	3.3	155	35.1	3.9	46.9	76.7	60.3	23.8
**TBDA2-6FDA-PI** [23]	223	390	12	47	8	285	32.5	4.0	35.6	48.7	27.9	23.8
**PIM-TB-PI-1** [39]	/	52.05	1.23	6.31	0.80	30.69	42.3	5.1	38.4	65.1	/	25.0
**PIM-TB-PI-2** [39]	/	122.34	3.05	12.20	2.44	59.49	40.1	4.0	24.4	50.1	/	19.5
**PI-TB-1** [37]	376	607	31	119	27	457	19	3.83	17	22	14	15
**PI-TB-2** [37]	86	134	2.5	14	2.1	55	53	5.48	26	64	41	22
**Polycarbonate** [40]	/	15.3	0.267	1.81	0.374	8.8	57.3	6.8	23.5	40.9	/	33.0
**Cellulose acetate** [41]	/	15.5	0.23	1.46	0.20	6.6	67.4	6.3	33	77.5	/	28.7
**Matrimid 5218** [8]	/	18	0.32	2.1	0.28	10	56	6.6	36	64	/	31
**Polysulfone** [42]	13	14	0.25	1.4	0.25	5.6	56	5.6	22.4	56	52	22.4

Unit mentioned: Barrer indicates 10^−10^ cm^3^ (STP)·cm/(cm^2^·s·cmHg).

**Table 5:** Diffusion coefficient (*D*) and solubility coefficient (*S*) for different gas species of **TBPI** and *m*-**MTBPI** membranes.

Samples	*D* (×10^−8^ cm^2^/s)				*S* (×10^−2^ cm^3^/cm^3^·cm·Hg^−1^)			
	N_2_	O_2_	CH_4_	CO_2_	N_2_	O_2_	CH_4_	CO_2_
**TBPI**	0.663	2.63	0.106	0.975	0.950	1.17	3.04	14.0
*m*-**MTBPI**	1.04	5.38	0.217	2.35	1.17	1.30	3.76	14.6

**Table 6:** Diffusion selectivity (*α*_D_) and solubility selectivity (*α*_S_) of **TBPI** and *m*-**MTBPI** membranes for different gas pairs.

Samples	*α*_D_	*α*_S_	*α*_D_	*α*_S_	*α*_D_	*α*_S_
	CO_2_/CH_4_	CO_2_/CH_4_	O_2_/N_2_	O_2_/N_2_	CO_2_/N_2_	CO_2_/N_2_
**TBPI**	9.20	4.61	3.97	1.23	1.47	14.74
*m*-**MTBPI**	10.83	3.88	5.17	1.11	2.26	12.48

**Table 7:** The mixed-gas permeability and selectivity of the *m*-**MTBPI** membrane tested at the pressure range of 2–20 bar and 35 °C.

Gas Species	Pressure	*P* (CO_2_) (Barrer)	*P* (CH_4_) (Barrer)	CO_2_/CH_4_ Selectivity
**Pure gas**	2 bar	34.4	0.820	42
**Mixed gas**	2 bar	38.7	0.773	50
	5 bar	30.2	0.656	46
	10 bar	27.3	0.660	41
	15 bar	25.9	0.721	36
	20 bar	25.0	0.764	33

## Data Availability

The raw data supporting the conclusions of this article will be made available by the authors upon request.

## Supplementary Materials

The following supporting information can be downloaded at: https://www.mdpi.com/article/10.3390/polym17040524/s1, Experimental section, Figure S1: Digital images of **TBPI** (a) and *m*-**MTBPI** (b) membranes; Figure S2: FT-IR spectra of (a) **BIDA** diamine and (b) **TBPI** polymer; Figure S3: ^1^H-NMR spectra of (a) **BIDA** diamine and (b) **TBPI** polymer in DMSO-*d*_6_. The asterisk indicates solvent and moisture residuals or the *H*-grease signal; Figure S4: GPC traces of the two **TB**-based polyimides; Figure S5: The overall gas separation performance of **TBPI**s and their unpretreated control group relevant to the 1991 and 2008 Robeson upper bound for (a) CO_2_/CH_4_ gas pair, (b) H_2_/CH_4_ gas pair, (c) H_2_/N_2_ gas pair, and (d) O_2_/N_2_ gas pairs. *Note*: Dark grey line and red line respectively indicates the 1991 and 2008 Robeson upper bound. **TBPI** and *m*-**MTBPI** represents the Soxhlet extracted PIs, while **Un-TBPI** and **Un**-*m*-**MTBPI** represents their corresponding unpretreated control groups.
