# Supplementary material for: Tröger’s Base Polyimide Membranes with Enhanced Mechanical Robustness for Gas Separation
Source: Polymers (Basel). 2025 Feb 18;17(4):524. doi: 10.3390/polym17040524 (PMC11859751; doi:10.3390/polym17040524)
Supplement: Supplementary file 1 [file polymers-17-00524-s001.zip › polymers-3441128-supplementary.pdf]

Supplementary Materials

Tröger's Base Polyimide Membranes with Enhanced Mechanical Robustness for Gas Separation

Xingfeng Lei,[1,2,3*] Zixiang Zhang,[1,2,3] Yuyang Xiao,[1,2,3] Qinyu Yu,[1,2,3] Yewei Liu,[1,2,3] Xiaohua Ma,[4] Qiuyu Zhang[1,2,3]

[1]Xi'an Key Laboratory of Functional Organic Porous Materials, School of Chemistry and Chemical Engineering, Northwestern Polytechnical University, Xi'an, 710072, China.

[2]Key Laboratory of Special Functional and Smart Polymer Materials of Ministry of Industry and Information Technology, School of Chemistry and Chemical Engineering, Northwestern Polytechnical University, Xi'an, 710072, China.

[3]Key Laboratory of Material Physics and Chemistry under Extraordinary Conditions of Ministry of Education, School of Chemistry and Chemical Engineering, Northwestern Polytechnical University, Xi'an, 710072, China.

[4]State Key Laboratory of Separation Membranes and Membrane Processes, National Center for International Joint Research on Membrane Science and Technology, Tiangong University, Tianjin, 300387, China.

[*]Correspondence: leifeng@nwpu.edu.cn; Tel.: +86-029-88431653.

Experiment section

Materials

2,2′-Bis(3,4-dicarboxy-phenyl) hexafluoro propane dianhydride (**6FDA**), 4,4'-diaminodiphenyl ether (**ODA**), 2-methyl-4,4'-diaminodiphenyl ether (*m*-**MODA**), N,N-dimethylacetamide (DMAc), polyformaldehyde, trifluoroacetic acid (TFA), dichloromethane (DCM), trichloromethane, methanol and ammonium hydroxide are purchased from J&K Scientific Ltd, China, and were directly used as received unless otherwise stated. Deionized water was used in all the experimental process.

Instrument and methods

Molecular weight and its polydispersity of the resulting polymers were tested by Waters 1515 gel permeation chromatography (GPC). THF was used as eluent at a flow rate of 0.5 $mL\cdot min^{-1}$ and polystyrenes were used as the external standard.

Fourier transform infrared (FT-IR) spectra were collected by Bruker Tensor 27 Fourier transform infrared spectrometer. KBr pellets were used as sample holder.

^{1}H-NMR and ^{13}C-NMR spectra were recorded by Bruker Avance 400 MHz nuclear magnetic resonance spectrometer. DMSO-d_6 were used as solvent and tetramethyl silane (TMS) were used as internal reference.

Thermogravimetric analyses (TGA) were carried out on a Mettler Toledo TGA2 synchronous thermal analyzer under nitrogen atmosphere at a heating rate of 10 $°C\cdot min^{-1}$ from room temperature to 800 °C.

Wide-angle X-ray diffraction (WAXD) measurements were carried out on a Shimadzu XRD700 X-ray diffractometer with cooper radiation (λ = 1.5406 Å) at a scanning rate of 3° per minute. The values of *d*-spacing were calculated according to Bragg's law ($n\lambda = 2d\cdot\sin\theta$).

Dynamic mechanical analysis (DMA) was carried out on a TA DMA850 dynamic thermomechanical analyzer to determine the glass transition temperature of the resulting polymers. The test membranes were cut into 30 × 5 mm^2 strips and then they were subject to a programmed heating rate of 2 °C·min^{-1} at a frequency of 1 Hz from 30 °C to 350 °C in a tensile mode. The glass transition temperature is regarded as the peak temperature of the internal loss curves.

Before the gas permeation test, all membranes were extracted by using an Soxhlet apparatus with anhydrous methanol for 24 h to completely remove the solvent residuals. After Soxhlet extraction, the membranes were directly subject to ambient air drying for 24 h followed by vacuum degassing at 35 °C for 24 h and then the membranes underwent gas permeation tests. The pure gas permeability (*P*) of the two membranes were tested on a TGM-1 gas permeation system at 2 atm and 35 °C by the constant-volume/variable-pressure time-lag method. The diameters and thickness of the membranes were around 1.5 cm and ~55 μm, respectively. Three individual membranes were tested for each sample, and the results were reported on average. *P* was determined by the following equation:

$$P = D \times S = \frac{V_{\mathrm{d}} l}{AT p_{\mathrm{up}} \times 0.278} \times \frac{dp}{dt} \times 10^{10} \qquad \text{(Eq. S1)}$$

where *P* is the pure gas permeability (barrer); p_{up} is the upstream pressure (mmHg); d*p*/d*t* is the increase rate of pressure in the downstream chamber at steady state; V_d is the calibrated permeate volume; *l* is the thickness of the membrane; *A* is the effective membrane area and *T* is the measurement temperature. The diffusion coefficient (*D*) was calculated from equation $D = l^2/6\theta$, where θ is the time lag of the permeability measurement. The solubility coefficient (*S*) was calculated from $S = P/D$. The mixed-gas separation measurement on membranes was also carried out using the constant-volume/variable-pressure time-lag method at 35 °C. Binary CO_2/CH_4 (50/50 in partial

pressure) and O_2/N_2 (22/78 in partial pressure) gas mixture was used as feed gas at the upstream pressure of 2-30 bar. The retention flow was controlled by a mass flow meter (MFC) with the stage cut of 0.01 to keep the component ratio of the feeding mixed gas to be constant. The permeated gas was cumulated in the downstream volume (24.59 mL) till ~40 torr, which was then sucked into a constant volume loop (2 mL) pre-installed of a six-way valve in an EWAI GC-4000A GC instrument, then the gas was sent to the analyzing column of GC and the components were identified by a thermal conductivity detector (TCD). The gas concentration of each component was analyzed by their areas in GC (GC-4000A, China) and corrected by a calibration factor (the area ratio of same amount of gases). Their permeabilities were calculated according to the following equation:

$$P_{CO_2} = \frac{y_{CO_2} \times V \times l}{x_{CO_2} \times P_{up} \times T \times 0.278 \times A} \times \frac{dp}{dt} \times 10^{10} \quad \text{(Eq. S2)}$$

$$P_{CH_4} = \frac{y_{CH_4} \times V \times l}{x_{CH_4} \times P_{up} \times T \times 0.278 \times A} \times \frac{dp}{dt} \times 10^{10} \quad \text{(Eq. S3)}$$

$$P_{O_2} = \frac{y_{O_2} \times V \times l}{x_{O_2} \times P_{up} \times T \times 0.278 \times A} \times \frac{dp}{dt} \times 10^{10} \quad \text{(Eq. S4)}$$

$$P_{N_2} = \frac{y_{N_2} \times V \times l}{x_{N_2} \times P_{up} \times T \times 0.278 \times A} \times \frac{dp}{dt} \times 10^{10} \quad \text{(Eq. S5)}$$

Where x_{CO2}, x_{CH4}, x_{O2} and x_{N2} are the feeding component ratio of CO_2, CH_4, O_2 and N_2, y_{CO2}, y_{CH4}, y_{O2} and y_{N2} are the permeate gas concentration of CO_2, CH_4, O_2 and N_2, respectively. P_{up} is the pressure of the upstream feeding gas. The selectivity of CO_2/CH_4 and O_2/N_2 can be calculated as follows:

$$\alpha_{CO_2/CH_4} = \frac{y_{CO_2}/y_{CH_4}}{x_{CO_2}/x_{CH_4}} \quad \text{(Eq. S6)}$$

$$\alpha_{O_2/N_2} = \frac{y_{O_2}/y_{N_2}}{x_{O_2}/x_{N_2}} \quad \text{(Eq. S7)}$$

Nitrogen physisorption isotherms were recorded by using a Tristar II 3020 apparatus coupled with Horvath-Kawazoe (H-K) method at 77 K for carbon slit pore

geometry provided by ASAP 2020 version 4.02 software. Before nitrogen sorption experiments, all samples were degassed under high vacuum at 100 °C for 24 h. According to the single-gas adsorption data, the apparent surface area of the resulting polyimides was calculated according to multipoint Brunauer-Emmett-Teller (BET) analysis.

Mechanical properties (ultimate tensile strength, elongation at break, and tensile modulus) were tested following ASTM D882-88 standard on rectangular membrane strips ($L \times W = 9 \times 4$ mm^2) by utilizing a CMT 4103 universal tensile testing instrument at room temperature. The tensile modulus is taken as the initial slope of the stress-strain curves. The mechanical properties were recorded as average values on at least five samples. The static toughness was determined by integrating the area under the stress-strain curves.

Characterizations

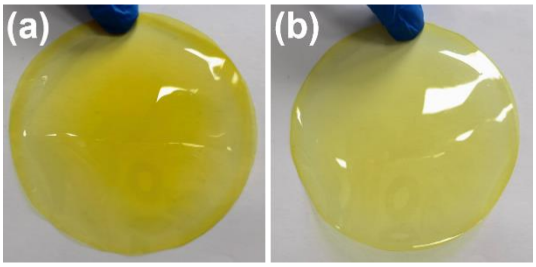

Figure S1. Digital images of **TBPI** (a) and *m*-**MTBPI** (b) membranes.

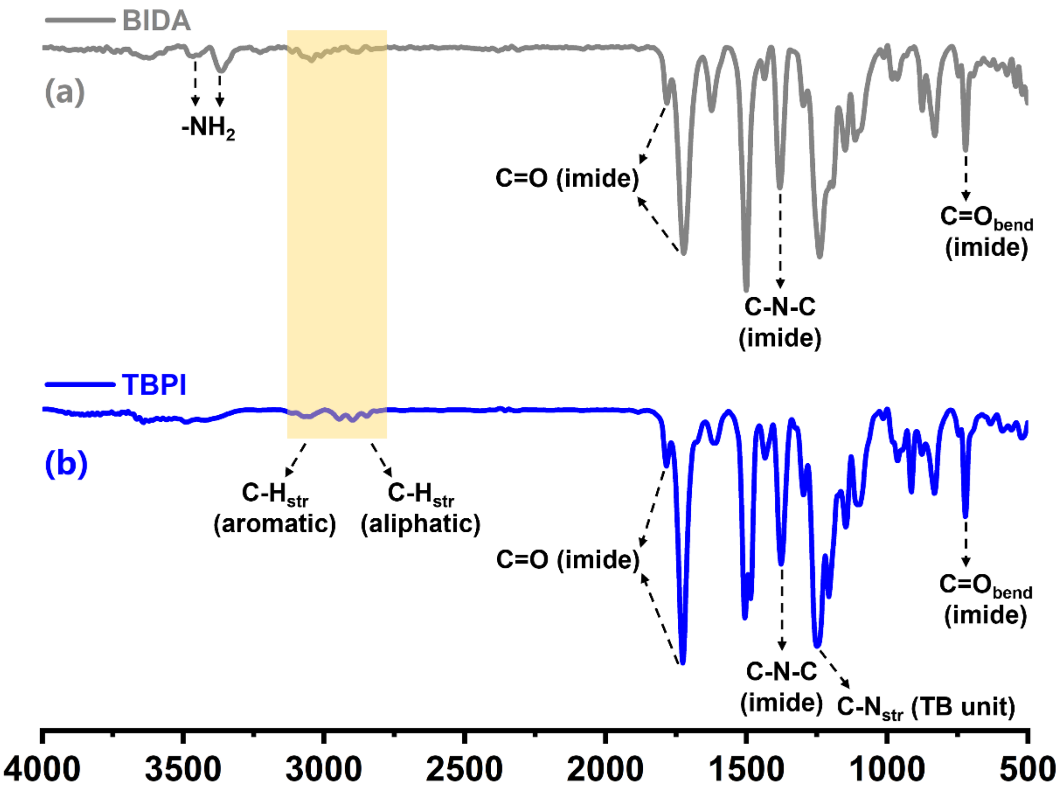

Figure S2. FT-IR spectra of (a) **BIDA** diamine and (b) **TBPI** polymer.

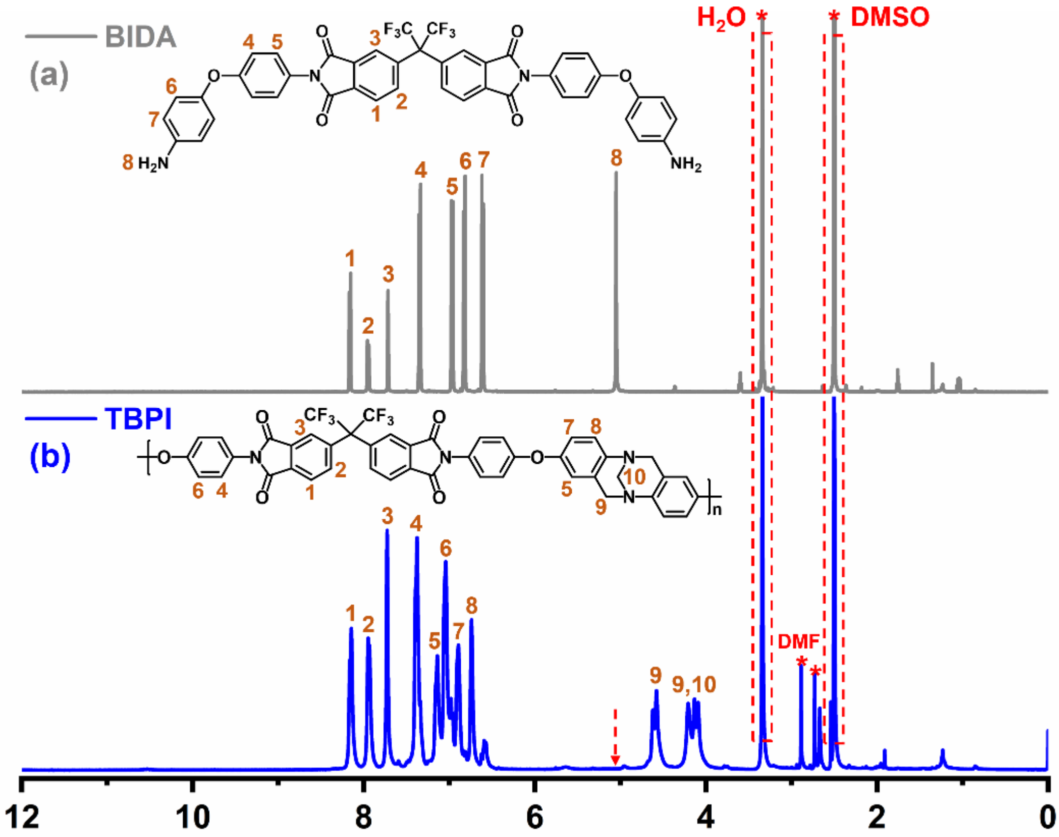

Figure S3. ^{1}H-NMR spectra of (a) **BIDA** diamine and (b) **TBPI** polymer in DMSO-d_6. The asterisk indicates solvent and moisture residuals or *H*-grease signal.

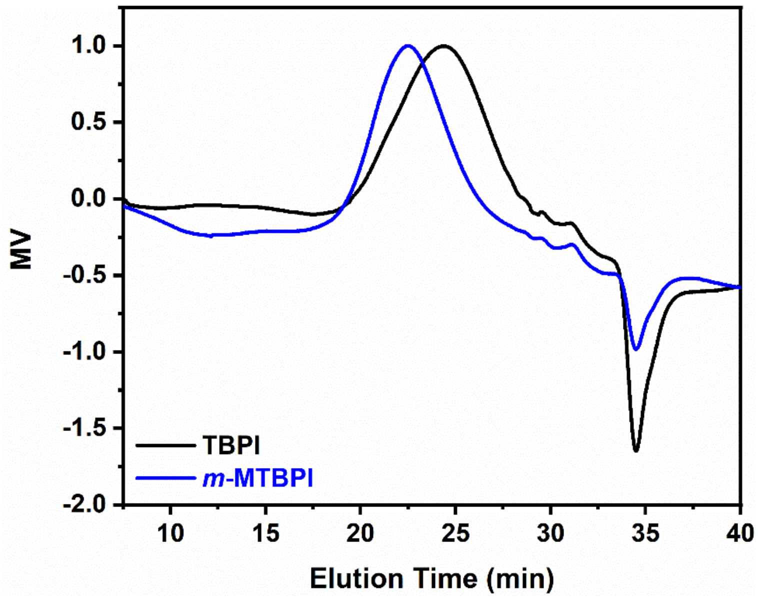

Figure S4. GPC traces of the two **TB**-based polyimides.

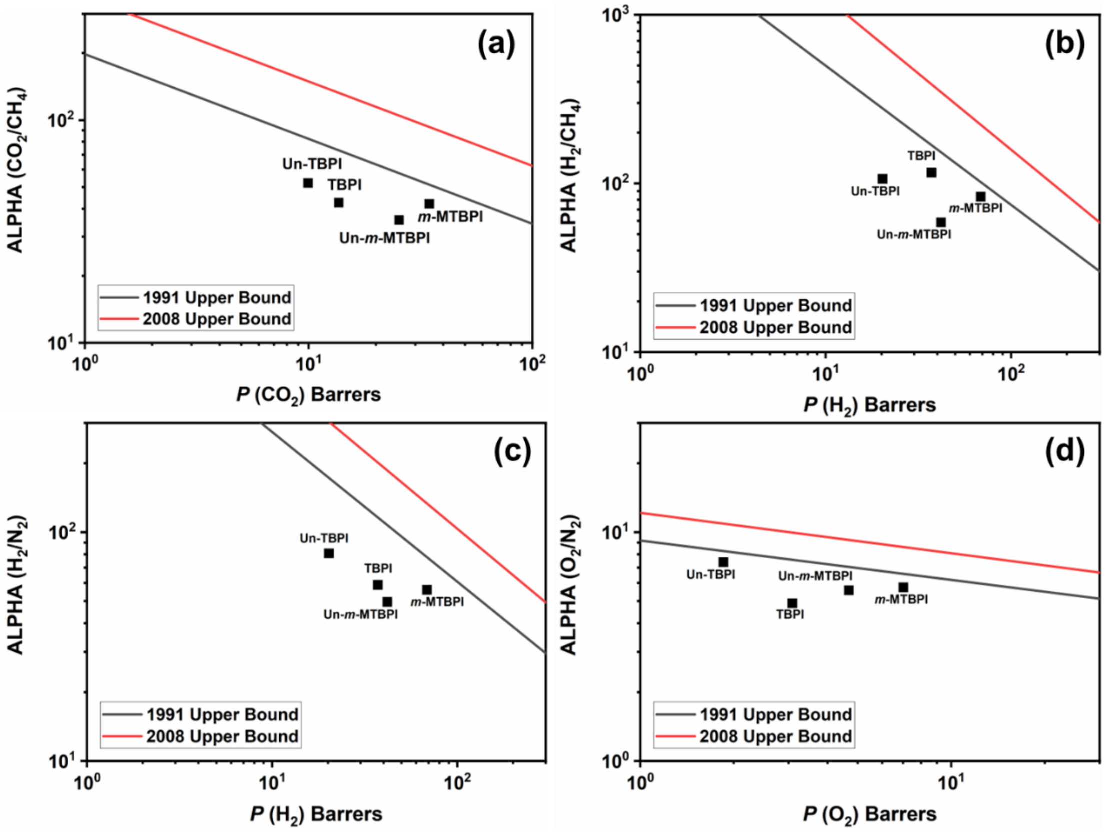

Figure S5. The overall gas separation performance of **TBPI**s and their unpretreated control group relevant to the 1991 and 2008 Robeson upper bound for (a) CO_2/CH_4 gas pair, (b) H_2/CH_4 gas pair, (c) H_2/N_2 gas pair, and (d) O_2/N_2 gas pairs. *Note*: Dark grey line and red line respectively indicates the 1991 and 2008 Robeson upper bound. **TBPI** and *m*-**MTBPI** represents the Soxhlet extracted PIs, while **Un-TBPI** and **Un-*m*-MTBPI** represents their corresponding unpretreated control groups.
